# Editorial: Reviews and perspectives in neuromorphic engineering: novel neuromorphic computing approaches

**DOI:** 10.3389/fnins.2024.1498684

**Published:** 2024-10-07

**Authors:** Pier Luigi Gentili, Siegfried Karg, Gyorgy Csaba, Konrad Szaciłowski

**Affiliations:** ^1^Department of Chemistry, Biology, and Biotechnology, Università degli Studi di Perugia, Perugia, Italy; ^2^IBM Research Europe (Zurich), Rüschlikon, Switzerland; ^3^Faculty for Information Technology and Bionics, Pázmány Péter Catholic University, Budapest, Hungary; ^4^Academic Centre for Materials and Nanotechnology, AGH University of Krakow, Kraków, Poland; ^5^Unconventional Computing Lab, University of the West of England, Bristol, United Kingdom

**Keywords:** Natural Computing, Oscillatory Neural Networks, graphene-based memristive devices, phase-change materials, optoelectronic devices, chemical reaction networks, synthetic biology, nanofluidic iontronics

In the XXI century, humanity is spurred to face global challenges: climate changes, pollution, shortage of clean water, food and energy. These challenges regard Complex Systems, such as the intertwined human societies, the world economy, urban areas, natural ecosystems, and the climate of the Earth (UN General Assembly, 2015; Martin, 2007; Harari, 2018; Gentili, 2021; Gentili et al., 2022). Whenever we deal with Complex Systems, we experience some limitations in their description, and in understanding and predicting their behavior. Such limitations outline the so-called Epistemological Complexity (Gentili, 2023). A limitation is due to Computational Complexity (Goldreich, 2008): many computational problems involving Complex Systems are solvable but intractable. Examples are (1) Practical problems, such as scheduling and the traveling salesman problem; (2) Fundamental science problems, such as the Schrödinger equation and protein folding; (3) Pattern recognition problems faced through machine learning algorithms. They are all exponential problems that become intractable when they have large dimensions: it is impossible to determine their exact solutions in a reasonable time, even if we use the fastest supercomputers in the world. A promising strategy to face Epistemological Complexity and, hence, Computational Complexity is Natural Computing (Rozenberg et al., 2012; Gentili, 2023). Natural Computing is an interdisciplinary research line that draws inspiration from nature to formulate (a) new algorithms, propose (b) new materials and architectures to compute, and (c) new methods and models to understand Complex Systems. Wealthy sources of inspiration for new computing architectures and algorithms are the human and animal brains. Their imitation has sparked the burgeoning field of neuromorphic engineering that promises to outperform conventional Artificial Intelligence (AI) algorithms and high energy-demanding hardware, offering a hopeful and optimistic outlook for the future of computing. Combining new algorithms, materials and architectures at the same time might be a complex task, but it may be the most promising route to Natural Computing (Maher et al., 2024). This Research Topic presents seven cutting-edge works in this field.

Among the many examples of analog computing, Rudner et al. highlight that Oscillatory Neural Networks (ONNs) are particularly alluring. Computing is carried out on the basis of the rich, complex, non-linear synchronization dynamics of an artificial neural network. Using the phase of oscillators enables a rich, robust, and parallel way of encoding of information, as it is often done in biological systems. Artificial ONNs often rely on some version of a Hebbian rule to define attractor states for the oscillators' phases. In their study, the authors, using computer simulations, demonstrate that a state-of-the-art machine learning method, namely Backpropagation Through Time, when applied to a circuit-level model of the ONN (based on resistively coupled ring oscillators), significantly enhances the computational power of the ONNs in recognizing various patterns.

Abernot et al. present possible algorithms and implementation of continual on-chip learning based on a digital ONN design for pattern recognition. They highlight that Hopfield Neural Network's unsupervised learning algorithms are compatible with ONN on-chip learning only if they satisfy two constraints on the weight matrix, the symmetry and the 0-diagonal, and two additional constraints on the learning algorithm, locality, and incrementality. The results of this work show that two unsupervised learning rules are compatible with ONN on-chip learning: Hebbian and Storkey. The proposed architecture takes advantage of a Processing System of a Zynq processor to implement the learning algorithms and Programmable Logic resources to implement the digital ONN.

Jiménez et al. describe an ONN implemented in a commercial CMOS technology to emulate the behavior of neural surrogates based on the phase-change VO_2_ material. VO_2_ undergoes metal-insulator transitions under given electrical stimuli. VO_2_ devices stand out for their hysteresis in the characteristic I–V curve, which enables compact low-power relaxation oscillators. The declared purpose of this work is to study in-depth the synchronization dynamics of relaxation oscillators similar to those that can be performed with VO_2_ devices. The fabricated circuit is very flexible since it allows programming the synapses to implement different ONNs, calibrating the frequency of the oscillators, or controlling their initialization. It uses differential oscillators and resistive synapses, equivalent to memristors. The ONN has been tested in its Associate Memory functionality.

Rajalekshmi et al. present a comprehensive analysis of the structural and design aspects of graphene-based Resistive Random Access Memory (RRAM) devices for their applications in in-memory and neural computing. Graphene-based RRAM devices are memristive systems with enhanced switching speed, retention time, endurance, and power consumption. Graphene assures additional performances, such as more substantial heat dissipation and chemical stability. Moreover, graphene provides more than two states to the memristive device, allowing the implementation of analog computing devices and storage.

El Srouji et al. proclaim that co-integrated photonic and electronic technologies are key to the future of neuromorphic computing. Biological neural networks are remarkably heterogeneous in terms of individual neuron dynamics and morphological structure. Such neural heterogeneity increases the sensitivity toward the complexity of behaviors and sensory modalities the brain must handle. An optoelectronic approach to neuromorphic computing is better suited to provide the interconnect bandwidths necessary to support the neuronal fan-in and fan-out required to model neural networks at biological scales while allowing for flexible and programmable neural dynamics.

Gentili et al. outline neuromorphic engineering in wetware, i.e., in a liquid solution, the peculiar phase supporting life. In wetware, three are the principal strategies to mimic some structural and functional features of the human brain. The first one, described also by Csizi and Lörtscher, relies on networks of chemical reactions: any solution containing reactive species can be compared to a neural network. Some reactions reproduce binary logic functions; others are appropriate for processing fuzzy logic. In the presence of strong non-linear interactions between the intermediate reactive species, some reactions exhibit bottom-up self-organization phenomena that reproduce the dynamics of real neurons and ONNs can be implemented. Such dynamic neural surrogates can communicate through electro-chemical and optical signals and become building blocks of feedforward and recurrent networks. When the molecules participating in the chemical reaction networks are biopolymers, such as DNA, RNA, and proteins, we enter the realm of synthetic biology, which constitutes the second strategy for developing neuromorphic engineering in wetware. The third strategy is nanofluidic iontronics, which represents the possibility of emulating neural networks through hybrid circuits made of solid nanochannels and electrically conductive ionic solutions.
